# Determinants of patient compliance with post-operative rehabilitation after lower limb orthopaedic surgery: a prospective study

**DOI:** 10.1186/s12891-025-09320-5

**Published:** 2025-11-22

**Authors:** Jonathan John Abraham, Sachin Kumar, Kiran Kumar Vedavyasa Acharya

**Affiliations:** Department of Orthopaedics, Kasturba Medical College, Manipal, Manipal Academy of Higher Education, Manipal, Karnataka 576 104 India

**Keywords:** Compliance, Physiotherapy, Rehabilitation, Post-operative, Socioeconomic, Health-care

## Abstract

**Objective:**

To assess patients’ compliance with post-operative rehabilitation protocols following orthopaedic lower limb surgeries, and to identify clinical, demographic, and socioeconomic factors leading to variability in compliance within and across different types of surgeries performed.

**Methods:**

This was a prospective study conducted in a tertiary healthcare hospital in South India. 240 patients who underwent orthopaedic lower limb surgery under the same unit, aged above 18 years, with cognitive capability to comprehend rehabilitation protocols, were included in the study. Patient compliance was assessed according to five parameters: adherence to appointments, weight-bearing activities, exercises, use of walking aids, and intake of medications. Patients’ compliance is scored dichotomously for a total score of five, followed by statistical evaluation (binary and multivariate regression analysis) to identify factors leading to variability in compliance.

**Results:**

53.7% patients were compliant (5/5). The highest compliance was with the intake of medications (91.7%), and the lowest was weight-bearing advice (75.4%). Statistical significance observed for compliance with the appointment date and exercises advised. Males (58.2%) and married individuals (58.3%) with (p value = 0.041 and 0.022, respectively) demonstrated higher compliance. 70.5% of patients in the government scheme in arthroscopy surgeries demonstrated non-compliance. 76.2% married individuals were compliant in the subgroup of other surgeries. 70% females who underwent arthroplasty demonstrated non-compliance. However, no independent predictor was statistically significant in the regression analysis.

**Conclusion:**

Understanding the factors leading to variation in compliance and tailoring the rehabilitation plan is essential to balance the intervention demands and the patient’s needs.

## Introduction

Orthopaedic lower limb surgeries require compliance with a structured rehabilitation programme to achieve optimal functional outcome. Abiding by the set of post-operative protocols is key to minimising recovery duration, reducing treatment cost, avoiding complications, overcoming hospital challenges, and achieving satisfactory long-term outcomes. Main patient-related factors resulting in non-compliance include: the barriers encountered, lack of positive feedback, and the degree of helplessness [1].

Existing literature focuses on identifying factors, such as demographic details like age, gender, marital status, socially disadvantaged individuals belonging to an ethnic minority group, unemployed individuals, and patients with a low socioeconomic background, during the treatment phase [1–5]. A cross-sectional study by Kattan et al. involving 359 post- orthopaedic surgery patients showed that 54% of the compliant participants were male, and 53.8% had attained a diploma or higher education. Younger patients (aged 18–35) were significantly more likely to skip physiotherapy sessions once they started feeling better or due to other commitments. Similarly, single individuals were more likely to miss sessions for the same reasons or due to inconvenient session timings [1]. Zelle et al. conducted a retrospective study to identify risk factors contributing to noncompliance with postoperative follow-up visits in orthopaedic trauma patients. The study results showed that 215 patients failed to attend at least one follow-up appointment. Regression analysis identified male gender, lack of commercial health insurance, government insurance, and smoking as significant risk factors for noncompliance [5].

Previous studies also looked into logistical drawbacks such as increased distance to the health centre, decreased level of education, and perceived injury severity [1, 4, 5]. These were possible predictors of non-compliance to follow-ups, resulting in pain, poor mobility, and health [1, 6]. Wharton. et al. 2014 conducted a study to evaluate patient compliance with postoperative follow-up. The results revealed that 183 patients (80%) adhered to their scheduled follow-up, while 46 (20%) did not. Younger age, male sex, and residence more. than 160.9 km from the hospital were significantly associated with decreased compliance. Another study by Jester et al. included 194 patients assessed knowledge of their post-injury weight-bearing status during their first clinic visit after hospital discharge, and results indicated that although 73% of patients participated in physical therapy after discharge, only 66% correctly identified their weight-bearing status [7].

However, the reason for the negative impact on post-operative rehabilitation varies depending on cultural background, socioeconomic context, and regional healthcare facility. While previous studies have identified predictors such as age, sex, education, marital status, and socioeconomic status, their relative influence is context-specific. In the regional locality, where disparities in healthcare access and awareness are prominent, the determinants of compliance remain underexplored. Patients might show lesser compliance with government based financial scheme due accessibility to health care facilities for routine follow-ups and financial constraints among the underprivileged who are eligible for government funding.

Furthermore, while demographic and socioeconomic factors are often studied, the role of financial schemes, geographic locality, and surgery type in influencing adherence requires further evaluation to provide more clarity on the inter-factorial relationship and their influence on patient compliance, which remains unexplored.

The study is designed to bridge that gap by evaluating the compliance with post-operative rehabilitation protocols after orthopaedic lower limb surgeries and exploring factors leading to variability in compliance within and across different types of surgeries performed.

## Materials and methods

### Study design and ethics approval

The prospective observational study was conducted between September 2023 and October 2024 in a tertiary health care hospital in South India. The study was approved by the Institutional Ethics Committee (IEC no: 322/2023) and followed the Strengthening the Reporting of Observational studies in Epidemiology (STROBE) guidelines for the research report [8]. Written informed consent was obtained.

### Participants

Inclusion Criteria

- patients above the age of 18 years.
- treated under the same orthopaedic unit.
- single injury to either lower limb.
- GCS of equal to or greater than 13 on post-operative day one of surgery.
- fit to comprehend the post-operative protocols advised.
- follow-up period after around one month.
- advised weight bearing and exercises, using walking aids, and prescribed medication at discharge.

Exclusion criteria

- non-consenting patients.
- those who developed new illnesses (examples: cardiovascular events, neurological deficits, infections) that could interfere with rehabilitation.
- patients treated/followed up elsewhere.

### Pre-discharge counselling

All patients in the study were counselled pre-discharge by the unit chief. Patients were advised on instructions to follow, made to perform the exercises along with gait training with a walking aid under supervision, and advised to obtain sufficient medica tions for one month till the appointment date before discharge.

While patients were encouraged to ensure medication purchase before discharge, compliance with medication intake was assessed at follow-up rather than at the time of discharge, thus respecting patient autonomy. Appointment dates were scheduled and communicated clearly to each patient, taking into account weekends and public holidays. Dressing changes and suture removal, where applicable, were integrated into the follow-up plan.

### Rehabilitation protocol

Patients were typically mobilised on the first or second post-operative day, depending on the surgery performed and clinical stability. Discharge occurred between the third and seventh post-operative day in most cases. A physiotherapist was involved in mobilisation and supervised initial exercise training in the ward.

### Scoring and outcome assessment

The scoring system was validated through review by three senior orthopaedic surgeons, establishing content validity. While no statistical reliability test was performed, consensus was obtained regarding its appropriateness for assessing compliance. The assessment was carried out by the unit chief to ensure uniformity, though this may have introduced observer bias, which is acknowledged as a limitation.

The unit chief assessed compliance with five parameters at one-month follow-up:

- Arrival of patient on scheduled appointment date.
- Adherence to weight-bearing.
- Usage of walking aids.
- Performing exercises advised.
- Intake of medications.

Each parameter was given a score of one if fulfilled or zero if not. A total score was calculated out of 5. Patients with a score of 5/5 were classified as compliant, and anything less was categorised as not compliant.

The following patient factors were recorded and analysed as subgroups according to different classification system:

Age (18–40, 41–60, and more than 60 years: categories selected to reflect functional, occupational, and geriatric differences) [9].

1. Sex (male, female) [10].
2. Educational status (no formal education, schooling, graduate, and post-graduate) [11].
3. Marital status (married, unmarried, and widowed) [12].
4. Occupation (none, daily wage worker, professional, self-employed) [13].
5. Body Mass Index (< 18.5, 18.5 kg/m2–22.9, >23 kg/m2) [14].
6. Geographic locality categorised as (rural, semi-urban, urban, metropolitan) [15].
7. Mechanism of injury (road traffic accident, fall from height, slip and fall, sports injuries, and others).
8. Comorbidities (nil or comorbid at the time of discharge).
9. Financial scheme (subsidised scheme, an insurance-based scheme, a government scheme, cash).

The surgeries were further subdivided into: arthroscopy, arthroplasty, and other orthopaedic procedures for subgroup analysis, since rehabilitation requirements differ substantially across these groups and patient compliance would vary based on the nature of the surgery performed.

### Statistical analysis

Data were entered into Microsoft Excel and analysed using SPSS software. Normality was checked with the Shapiro–Wilk and Kolmogorov–Smirnov tests. Categorical variables were expressed as proportions and percentages. Associations between compliance and patient variables were examined using chi-square tests. Binary logistic regression was conducted to explore independent predictors of compliance. A p-value < 0.05 was considered statistically significant.

## Results

A total of 240 patients were evaluated, 129 (53.7%) complied with the rehabilitation protocols, whereas 111 (46.3%) were non-compliant.

**Fig. 1:**
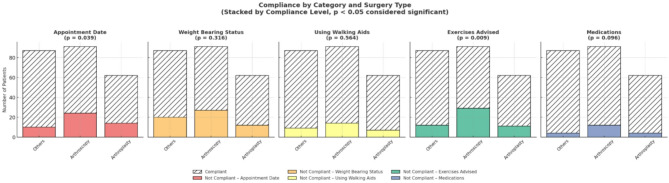
Compliance of patients across five parameters based on the surgery performed

Compliance across the five parameters (Fig. 1):

- Arrival on the advised appointment date: 80%.
- Adherence to weight bearing: 75.4% (lowest compliance).
- Usage of walking aid: 87.5%.
- Performing exercises advised: 78.3%.
- Intake of medications: 91.7% (highest compliance).

Statistical significance was observed for compliance with appointment date (*p* value = 0.039) and exercises advised (*p* value = 0.009).

Among the different factors analysed (Table 1.), compliance was higher among males (58.2%; *p* value = 0.041) than among females. Married patients exhibited more compliance (58.3%) than unmarried or widowed patients (*p* = 0.022). The younger age group of patients showed higher compliance (59.1%). Higher levels of educational categories correlated with higher compliance among patients. Postgraduates with a compliance of 70.6% had the highest level of compliance. Road traffic accidents and sports injuries had higher compliance rates, with 64.8% and 63% of patients in each subgroup. Patients with no comorbidities at discharge showed slightly better compliance (55.6%). Patients residing in the rural and metropolitan areas showed lower levels of compliance, 42.9% in each category. Patients making cash payments had the highest compliance (63.9%) compared with other schemes. Factors such as occupation and body mass index had an unequal data distribution among the subgroups, making the analysis unreliable.

**Table 1.** The compliance in patients across subgroups of different factors

Variable	Category	n=240	Compliance		Total	P value
			Not compliant	Compliant		
**Age**	18–40 years	n	47	68	115	0.265
		%	40.90%	59.10%	100.00%	
	41–60 years	n	34	34	68	
		%	50.00%	50.00%	100.00%	
	> 60 years	n	30	27	57	
		%	52.60%	47.40%	100.00%	
**Sex**	Male	n	69	96	165	0.041
		%	41.80%	58.20%	100.00%	
	Female	n	42	33	75	
		%	56.00%	44.00%	100.00%	
**Educational status**	No formal education	n	28	25	53	0.171
		%	52.80%	47.20%	100.00%	
	Schooling	n	40	42	82	
		%	48.80%	51.20%	100.00%	
	Graduate	n	33	38	71	
		%	46.50%	53.50%	100.00%	
	Postgraduate	n	10	24	34	
		%	29.40%	70.60%	100.00%	
**Marital status**	Unmarried	n	27	30	57	0.022
		%	47.40%	52.60%	100.00%	
	Married	n	65	91	156	
		%	41.70%	58.30%	100.00%	
	Widowed	n	19	8	27	
		%	70.40%	29.60%	100.00%	
**Occupation**	None	n	67	65	132	0.230
		%	50.80%	49.20%	100.00%	
	Daily wage worker	n	6	12	18	
		%	33.30%	66.70%	100.00%	
	Professional	n	24	39	63	
		%	38.10%	61.90%	100.00%	
	Seld-employed	n	14	13	27	
		%	51.90%	48.10%	100.00%	
**Body Mass Index**	< 18.5 kg/m2	n	5	3	8	0.385
		%	62.50%	37.50%	100.00%	
	18.5 kg/m2- 22.9 kg/m2	n	32	46	78	
		%	41.00%	59.00%	100.00%	
	> 23 kg/m2	n	74	80	154	
		%	48.10%	51.90%	100.00%	
**Mechanism of injury**	Road traffic accident	n	25	46	71	0.087
		%	35.20%	64.80%	100.00%	
	Fall from height	n	4	5	9	
		%	44.40%	55.60%	100.00%	
	Slip and fall	n	31	23	54	
		%	57.40%	42.60%	100.00%	
	Sports injuries	n	10	17	27	
		%	37.00%	63.00%	100.00%	
	Others	n	41	38	79	
		%	51.90%	48.10%	100.00%	
**Comorbidities**	Nil	n	72	90	162	0.419
		%	44.40%	55.60%	100.00%	
	Comorbidatthe time of discharge	n	39	39	78	
		%	50.00%	50.00%	100.00%	
	Rural	n	20	15	35	0.333
		%	57.10%	42.90%	100.00%	
**Geographic Locality**	Semi-urban	n	48	69	117	
		%	41.00%	59.00%	100.00%	
	Urban	n	39	42	81	
		%	48.10%	51.90%	100.00%	
	Metropolitan	n	4	3	7	
		%	57.10%	42.90%	100.00%	
**Financial Scheme**	Subsidized	n	16	14	30	0.065
		%	53.30%	46.70%	100.00%	
	Insurance based	n	12	18	30	
		%	40.00%	60.00%	100.00%	
	Government	n	53	44	97	
		%	54.60%	45.40%	100.00%	
	Cash	n	30	53	83	
		%	36.10%	63.90%	100.00%	

**Fig. 2:**
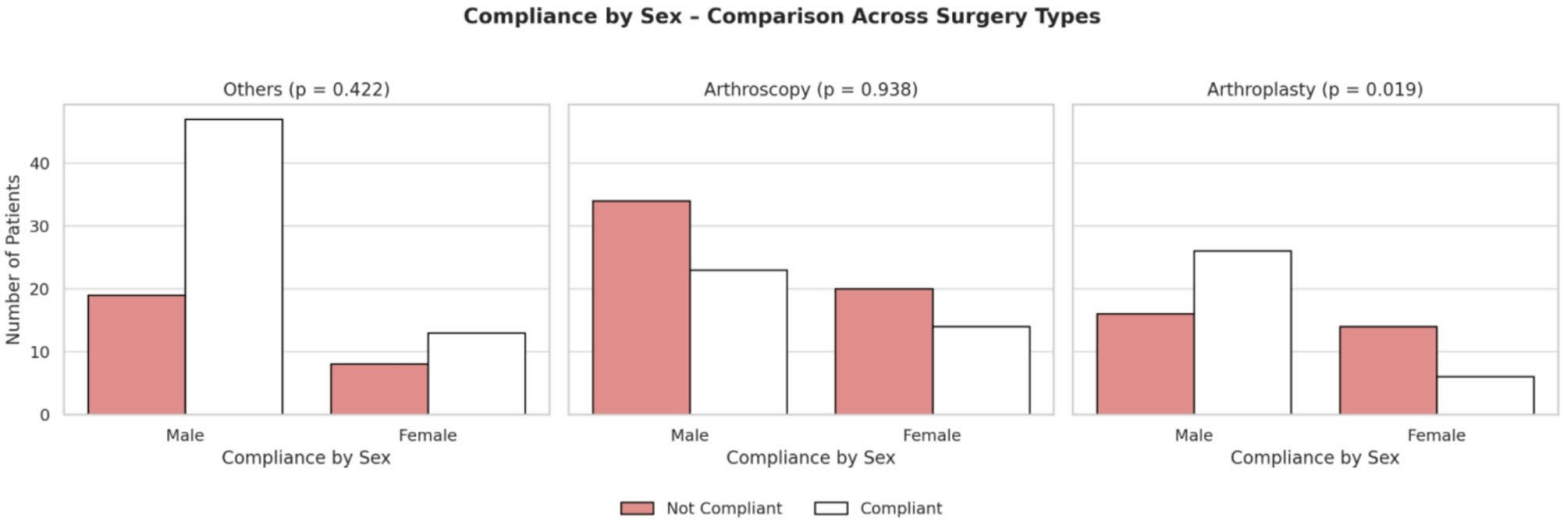
Compliance according to sex across surgeries performed

**Fig. 3:**
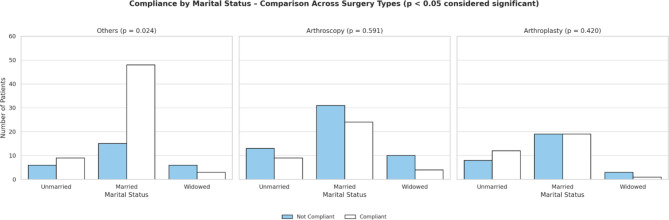
Compliance according to marital status across surgeries performed

Among the 240 patients, most patients belonged to the category of arthroscopy surgeries (37.9%), followed by other orthopaedic surgeries (36.3%), and arthroplasty surgeries (25.8%). Compliance observed in subgroups of surgeries: Arthroscopy surgeries demonstrated the highest non-compliance across surgeries performed. Patients under the government scheme, particularly in the arthroscopy subgroup, had the highest non- compliance (70.5%; *p* = 0.037). Females in arthroplasty surgeries demonstrated 70% non- compliance (*p* = 0.019) (Fig. 2). Married patients in other orthopaedic surgeries demonstrated the highest compliance (76.2%; *p* = 0.024) (Fig. 3).

Despite associations observed in bivariate analysis, no independent variable proved to predict compliance in regression analysis (Table 2). This suggests an interplay of multiple demographic, socioeconomic, and clinical factors.

**Table 2:** Regression analysis of factors

Variable	*p*-value	Odds ratio	95% confidence interval for Odds ratio	
			Lower	Upper
Age	0.87	1.047	0.602	1.823
Sex	0.216	0.64	0.316	1.298
Educational status	0.371	1.203	0.803	1.802
Marital status	0.284	0.791	0.515	1.215
Occupation	0.619	0.929	0.696	1.241
BMI	0.457	0.832	0.512	1.352
Mechanism of injury	0.231	0.902	0.762	1.068
Comorbidities	0.606	1.207	0.59	2.467
Geographic locality	0.941	0.986	0.685	1.419
Financial scheme	0.082	1.275	0.969	1.677

## Discussion

In recent years, valuable thought processes have been invested in shaping targeted patient care and awareness to elevate the quality of the healthcare system. Compliance with the treatment plan is both the result and indicator of the quality of care experi enced by the patients. This study demonstrates the sociodemographic factors pivotal in understanding compliance patterns with post-operative rehabilitation protocols (Table 1.). The importance of effective rehabilitation is understood with the enhanced recovery after surgery protocol, demonstrating less post-operative pain and complications, decreased mortality, and hospital readmission rates [16].

Medication adherence was highest in our cohort (91.7%). This is consistent with Kattan et al. (2023), who reported medication adherence above 85% among orthopaedic patients in Saudi Arabia [1]. Similarly, Bender et al. (2024) observed high adherence to prescribed medications among U.S. orthopaedic surgery patients [6].

Exercise adherence in our study was 78.3%, aligning with Campbell et al. (2001), who found compliance with physiotherapy exercises in knee osteoarthritis to be around 70% [17]. Comparable rates of 65–75% have also been reported in systematic reviews of adherence to physical therapy [3]. Weight-bearing adherence was lowest in our study (75.4%), a similar finding noted by Zelle et al. (2015), who documented significant loss to follow-up and non- compliance with mobility restrictions in orthopaedic trauma patients [5]. Jester et al. (2021) also noted that nearly one-third of orthopaedic trauma patients deviated from prescribed mobility instructions [7]. These findings confirm that active rehabilitation tasks, such as exercises and weight-bearing, are more challenging for patients than passive tasks like medication intake.

In this study, younger patients (59.1% in 18–40 years) and patients with higher education (postgraduates- 70.6%) directly correlate with better compliance postoperatively. It can be due to social norms of older people becoming more dependent on caretakers, and mobility issues due to poor geriatric care and health maintenance. Kattan et al. observed that younger individuals and educated patients were less compliant and had higher chances of missing appointments, attributed to more responsibilities, busier lifestyle, and poorer work- life balance [1]. Mathes et al. and Bender et al. observed that higher education correlated with more employment and contributed to better compliance, like in this study [3, 6]. However, a relationship between marital status and compliance was not found [3]. On the contrary, our research observed that married patients showed higher compliance (58.3%) than unmarried (52.6%) and widowed patients(29.6%), possibly due to a lack of social support and psychosocial factors (Fig. 3).

Cash-paying patients had the highest compliance (63.9%), while government-funded patients were least compliant (45.4%), possibly due to the lack of accessibility to health care facilities for routine follow-ups and financial constraints among the underprivileged, while patients paying cash, perhaps, could have the motivation to put in maximum effort to aid in betterment. Zelle et al. (2015) reported similar findings, with patients on public assistance more likely to be lost to follow-up. However, the same research could not find significant observations on the mechanism of injury and compliance [5]. In contrast, in this study, patients involved in road traffic accidents (64.8%) and sports injuries (63.0%) showed higher compliance than patients involved in slip and fall injuries (42.6%), possibly due to the perceived severity of injury among patients involved in high-velocity injuries. The age groups involved in the mechanism are also important, as sports injuries are common among the younger population, who have adequate social independence to undergo recommended rehabilitation protocols. At the same time, slip and fall is more common among older adults, who are socially dependent on day-to-day activities.

Higher compliance was observed among males (58.2%) compared to females (44%), indicating the sex-based differences between both genders in seeking health care in their respective societies and support systems. Zester et al. and Bender et al. demonstrated that males showed patterns of non-compliance and developed the feeling to get better post- surgery [6, 7].

In our study, the patient’s comorbidities at discharge did not show significant variation in compliance (No comorbidities- 55.6% vs. Comorbid at discharge- 50.0%).

Campbell et al. suggested that initial compliance with treatment was high in patients with comorbidities, due to the presumed severity of symptoms [17].

Geographic location influenced compliance: rural and metropolitan patients (42.9% each) were least compliant. Subjects at both ends of the spectrum demonstrated low compliance, possibly due to the accessibility to health care centres and financial constraints while residing in rural areas. In contrast, in urban towns, patients are exposed to a busier lifestyle, where postoperative rehabilitation could seem challenging. Casp et al. (2017) reported that 12% of patients cited distance and geographic barriers as reasons for poor follow-up in orthopaedic trauma [4].

In most cases, patients undergoing arthroscopic surgery exhibited poor compliance, possibly due to the underestimation of the surgery performed with the smaller incision and the elective nature (Fig. 1). A high majority of patients (70.5%) in arthroscopy surgery, who are government-funded, could be the reason for the increased non-compliance. Focused improvement in terms of rehabilitation for patients undergoing arthroscopy surgeries with educational support and awareness could be measures that enhance surgery outcomes. Females undergoing arthroplasty (70%) were less compliant than males, requiring close monitoring and frequent follow-ups (Fig. 2). Married patients undergoing “other surgeries” were the most compliant (76.2%), highlighting the protective role of social support.

The study highlights the multifactorial inter-relationship of factors influencing patients’ compliance after orthopaedic lower limb surgeries, suggesting that no variable alone correlates with the outcome, as reflected in the multivariate regression analysis (Table 2). Addressing accessibility to healthcare among the underprivileged population and programming patient-specific, focused intervention plans into the management protocols adds to healthcare quality and helps the physician treat the patient with the disease, not just the disease itself.

Future scope of standardising health-care should focus on integrating these variables into providing community programmed rehabilitation support, a multidisciplinary approach for post-operative care, digital care tools for structured follow-up, and specific group-based rehabilitation sessions.

The study proves to have many strengths, such as its prospective design, uniform unit-based counselling, and subgroup analysis.

However, the study has limitations as well, such as categorical scoring (not capturing frequency or quality of rehabilitation), single-assessor bias, and a short one-month follow- up. Categorisation of continuous variables such as age and BMI may have reduced statistical power. Furthermore, although associations were observed, multivariate regression did not identify independent predictors, indicating that compliance is multifactorial. This should be considered a hypothesis for future research.

### Clinical messages

- Active parameters of rehabilitation (exercises advised and weight-bearing status) show lower compliance than passive parameters (medication intake).
- It’s an interplay between various factors after surgery, determining patients’ compliance with rehabilitation protocols post-surgery, accounting for physical, mental, and social dimensions.
- Identifying these factors during the perioperative period and incorporating a patient- specific treatment plan is essential for the surgeon to achieve optimum post-surgery results during rehabilitation.

## Data Availability

The datasets generated and/or analyzed during the current study are not publicly available but are available from the corresponding author on reasonable request.
